# Adapted Physical Activity to Ensure the Physical and Psychological Well-Being of COVID-19 Patients

**DOI:** 10.3390/jfmk6010013

**Published:** 2021-01-29

**Authors:** Grazia Maugeri, Giuseppe Musumeci

**Affiliations:** 1 Department of Biomedical and Biotechnological Sciences, Human, Histology and Movement Science Section, University of Catania, via S. Sofia 87, 95123 Catania, Italy; graziamaugeri@unict.it; 2 Research Center on Motor Activities (CRAM), University of Catania, via S. Sofia 87, 95123 Catania, Italy; 3 Department of Biology, Sbarro Institute for Cancer Research and Molecular Medicine, College of Science and Technology, Temple University, Philadelphia, PA 19122, USA

**Keywords:** COVID-19, prevention, physical activity, inactivity, home-based exercise, mental health, psychological well-being

## Abstract

The novel coronavirus disease 2019 (COVID-19) has been responsible for a global pandemic involving massive increases in the daily numbers of cases and deaths. Due to the emergency caused by the pandemic, huge efforts have been made to develop COVID-19 vaccines, the first of which were released in December 2020. Effective vaccines for COVID-19 are needed to protect the population, especially healthcare professionals and fragile individuals, such as older people or chronic-disease-affected patients. Physical exercise training generally has health benefits and assists in the prevention of several chronic diseases. Moreover, physical activity improves mental health by reducing anxiety, depression, and negative mood and improving self-esteem. Therefore, the present review aims to provide a detailed view of the literature, presenting updated evidence on the beneficial effects of adapted physical activity, based on personalized and tailor-made exercise, in preventing, treating, and counteracting the consequences of COVID-19.

## 1. Introduction

The social effects caused by the global spread and pandemic of SARS-CoV-2 is having unimaginable consequences that the world has never faced in past decades. After the WHO declared SARS-CoV-2 a health emergency, the world responded quickly to “flatten the curve” or limit the spread of the virus by banning travel and closing non-essential businesses and educational institutions, as well stopping all kinds of large gatherings. In this first phase of the pandemic, around half of the world’s population was under full or partial lockdown to limit the spread of the deadly virus [1]. The unprecedented restrictions prompted by the raging SARS CoV-2 pandemic halted a wide variety of economic activities throughout the world. Day by day, the need for essential healthcare equipment increased in parallel with the increase of infected patients and death tolls. More than 100 countries closed their borders and worldwide air travel demand plummeted just after the announcement of the pandemic by the WHO. This severely impacted the world’s supply chain and international trade [2]. Economists agreed that there would be an enormous negative impact on global economic development due to COVID-19, which would possibly plunge the world economy into a deep recession [3]. Therefore, after this initial lockdown period, a second phase started, involving the partial reopening of the economy. However, increasing cases and limited numbers of intensive care beds have caused public health authorities in Europe to re-impose temporary lockdowns.

Global health authorities have been informed by epidemic and infectious disease specialists, who have faced the present health emergency by taking cues from past epidemics. However, we now know that the story of COVID-19 cannot be compared to past epidemics. Besides the high direct mortality for such a contagious acute disease, COVID-19 has placed extreme pressure on healthcare systems, altering access to health services of patients living with other pathologies, such as non-communicable diseases (NCDs). Moreover, such NCDs (e.g., diabetes mellitus, hypertension, cerebrovascular disease, coronary artery disease, chronic obstructive pulmonary disease) have been shown to predict poor prognosis in patients with COVID-19. SARS-CoV-2 and NCDs are clustering within social groups according to patterns of socioeconomic inequality that are deeply embedded in our societies. Limiting the harm caused by SARS-CoV-2 will demand that far greater attention is paid to NCDs and socioeconomic inequality than has previously been done. The aggregation of these diseases against a background of social and economic disparity aggravates the adverse effects of each separate disease. COVID-19 is not a pandemic or simple comorbidity—it is a syndemic [4]. Addressing COVID-19 means addressing hypertension, obesity, malnutrition, diabetes, cardiovascular and chronic respiratory diseases, cancer, and psychological and neurodegenerative disorders. Paying more attention to NCDs and socioeconomic inequality should be a strategic plan for both rich and poor nations in order to limit the harm caused by SARS-CoV-2.

The worldwide spread of SARS-CoV-2 infection has caused governments of various countries to take swift and unprecedented protective measures, including placing cities in lockdown and closing places where large gatherings would occur. Quarantine has radically changed the daily habits of the entire population, requiring people to practice “social distancing”. For example, in Italy, the Italian Ministry of Education (MIUR) decided to invest huge resources in special desks to promote higher interpersonal distance in classrooms to avoid the risk of infection [5]. Although such strategies have contained the COVID-19 outbreak, the prolonged self-isolation has deeply affected active lifestyles, leading healthy individuals and athletes to states of physical inactivity, with related consequences of hypomobility and inactivity-associated disorders, such as a reduction in maximal oxygen consumption (VO2max), endurance capacity, loss of muscle strength and mass, overweight, and decrease joint lubrification [6,7,8,9]. Just a few days of a sedentary lifestyle are sufficient to induce fiber denervation, insulin resistance, and low-grade systemic inflammation [10].

The positive effects of regular physical activity on general health are well known in the field of modern medicine. Physical activity counteracts cardiovascular vulnerability, inflammation, muscle atrophy, bone and cartilage loss or degeneration, and the reduction of aerobic capacity [11,12]. Physical exercise is also closely related to cognitive function and neurodegenerative disorders by inducing cellular and molecular processes underlying neurogenesis and synaptogenesis cascade, which enhance learning, memory, and brain plasticity [13,14]. These effects are extremely important, especially in light of new evidence showing brain damage among the consequences of COVID-19, including delirium, stroke, and brain inflammation. Moreover, adapted physical activity ameliorates one’s self-esteem and provides a sense of well-being by reducing the development of mental disorders [15]. In light of this evidence, the purpose of this narrative mini-review is to summarize the beneficial effects of the adapted physical activity performed before-, during-, and post-infection of COVID-19. To this end, four databases were used: PubMed, Scopus, Web of Science, and Google Scholar. The last search was conducted on 30 December 2020. The following keywords and combinations thereof were used: “exercise”, “physical activity”, “adapted physical activity”, “physical exercise”, “SARS-CoV-2”, “SARS CoV-2 pandemic”, “COVID-19 pandemic”. The initial study selection was performed via title and abstract screening. Duplicates were removed. The full texts of the selected articles were carefully read and analyzed in order to extract the appropriate data from each text.

## 2. The Beneficial Effects of Physical Activity before COVID-19 Infection

The development of COVID-19 is strictly linked to the interaction between SARS-CoV-2 and the host’s immune system. The virus affects the response of the immune system, leading to leukopenia with high levels of pro-inflammatory mediators. Several studies have shown that in mild cases of COVID-19, macrophages of pulmonary tissue are able to counteract SARS-CoV-2 and the innate and adaptive immune responses are able to fight viral replication. In contrast, severe cases of COVID-19 provoke a storm of pro-inflammatory cytokines and a lymphopenia state [16]. This “hyper inflammation” is characterized by aberrant pathogenic T cells and inflammatory monocytes, which are rapidly activated and produce a large number of cytokines, thus inducing the inflammatory storm [16]. Moreover, some COVID-19-affected patients have developed acute demyelinating encephalomyelitis (ADEM), without showing respiratory symptoms [17,18], and in some cases Guillan-Barrè syndrome has been diagnosed, characterized by nerve damage [19]. The SARS-CoV-2 virus is usually not present in patients’ cerebrospinal brain fluid. Therefore, it is possible to speculate that brain inflammation is caused by the immune system and that the neurological complications of COVID-19 might be provoked by the deregulated immune response rather than the virus itself [20]. The role of physical exercise in improving immune response has largely been demonstrated. Moderate and adapted physical activity increases the anti-inflammatory cytokines, immunoglobulins, and immune cells in circulation, as well as the anti-pathogenic activity of macrophages [21]. In this way, physical exercise may cause reductions of the burden of pathogen and the abnormal inflammatory cells that damage the lungs [22]. Interestingly, COVID-19-affected patients have reported higher levels of cytokines, such as TNF-α, IFN-γ, IL-1β, and IL-6, as compared to healthy subjects [23,24]. Moreover, the expression levels of these pro-inflammatory cytokines to be appeared directly related to the severity of the patient’s condition, confirming that the activation of the inflammatory process is linked to disease severity [23,24].

The implementation of the physical activity program could play a key role in counteracting the imbalance in antiviral immunity, protecting the individual against inflammation induced by COVID-19. According to WHO recommendations, all adults should undertake 150–300 min of moderate-intensity, 75–150 min of vigorous-intensity physical activity, or some equivalent combination of moderate-intensity and vigorous-intensity aerobic physical activity per week. Among children and adolescents, an average of 60 min/day of moderate–vigorous-intensity aerobic physical activity across the week provides health benefits. These guidelines recommend regular muscle-strengthening activity for all age groups [25]. The adapted physical activity comprises an exercise program designed in a personalized way in order to adapt to the physiological characteristics and the state of health of each subject. Its beneficial role in low-grade chronic inflammation was demonstrated in both the periphery and in the brain [26]. During its practices, stress hormones and microglia proliferation are decreased. Moreover, physical activity attenuates the release of proinflammatory cytokines through the modulation of anti-inflammatory cytokines, such as IL-1Ra, IL-6, and IL-10, as well as cytokine inhibitors, such as cortisol, prostaglandin E2, and soluble receptors against TNF and IL-2 [26]. Considering that adapted physical activity has shown several benefits for most chronic diseases and microbial infections with preventive and therapeutic effects, in the pre-infection phase, it may represent an important tool to prevent COVID-19 infection [27]. Furthermore, several pieces of evidence supported the direct relationship between exercise and psychological well-being. Individuals who practice regular physical activity ameliorate one’s self-esteem and provide a sense of well-being, leading to reduced depressive and anxiety symptoms [28,29]. This plethora of positive effects is due to the involvement of the hypothalamic–pituitary–adrenal (HPA) axis and the endogenous opioid system, both of which are implied in anxiety, stress, depression, and emotional responses [30,31]. In addition, regular exercise promotes the release of several trophic factors, including brain-derived neurotrophic factor (BDNF), which exerts a positive role in both anxiety and depressive disorders [32]. Quarantine and physical isolation measures may have had long-lasting and wide-ranging negative psychosocial impacts, which may have been amplified by a reduction in physical activity levels. Several works have demonstrated the negative impacts of decreased physical activity on psychological well-being [33,34,35,36]. In particular, a decrease in the amount of physical activity is associated with higher levels of perceived stress and anxiety [33]. A study performed on older adults showed that those who met the global recommendations on physical activeness had higher levels of resilience and lower levels of depressive symptoms [36]. The promotion of resilience during the COVID-19 pandemic is a crucial aspect for patients, considering that it is linked to positive emotions in stressful situations, locus of control, self-efficacy, optimism, and better quality of life (physical and psychological) [35]. In particular, Lesser and Nienhuis [33] reported that individuals who were more physically active showed greater mental health scores, whereas inactive subjects before the COVID-19 pandemic who became more active during the lockdown exhibited lower levels of anxiety. Moreover, a cross-national study between Germany, Italy, Russia, and Spain, showed that individuals with depression symptoms are at risk of developing a worse psychological condition during the current Covid-19 pandemic; instead, physical activity counteract such negative effects [37]. Interestingly, the profoundly negative impacts on psychological health and well-being in the population seem to be higher in females and young adults [32].

## 3. Adapted Physical Activity Program during COVID-19 Infection

**Figure 1:**
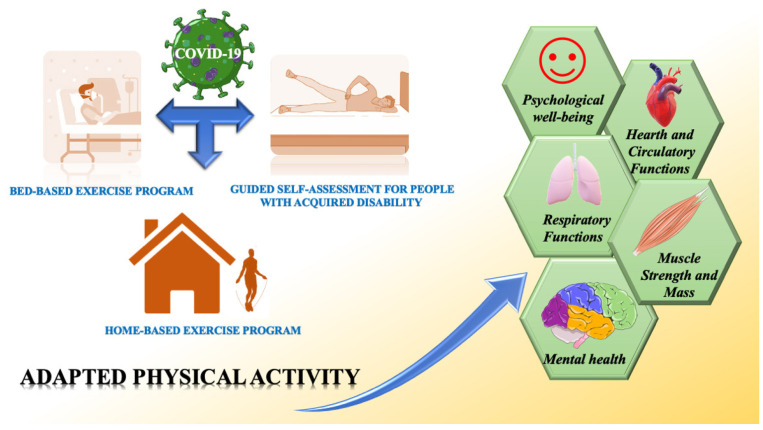
Health benefits from regular adapted physical activity in COVID-19-affected patients.

Considering the clinical characteristics of COVID-19, infected patients, who are compelled to rest in bed, are not able to perform normal activities of daily life or perform regular physical activity. Nevertheless, considering the multiple positive effects caused by exercise, adapted physical activity in all phases of recovery of patients (Figure 1) represents an important strategy to attenuate the decline in cognition function and to improve physical and psychological well-being in individuals affected by COVID-19. When treating patients—and given the intensive medical management involved for some COVID-19 patients, including prolonged protective lung ventilation, immobility, sedation, and treatment with neuromuscular blocking agents—in the acute phase it is possible to adopt only passive types of exercise performed by physiotherapists or kinesiologists, such as whole-body vibration (WBV) exercise and passive range-of-motion (pROM) exercises. In the post-acute phase, however, physiotherapists or kinesiologists can organize bed-based exercise programs (e.g., flexion and extension of the limbs and trunk) and assist patients to mobilize independently to stand-up and perform normal daily functions according to the Barthel index, such as washing, eating, and so on. Other adapted physical activities, comprising passive, active-assisted, active, or resisted joint range-of-motion exercises, are fundamental to restore and improve respiratory and cardiocirculatory functions, joint integrity, range-of-motion, muscle strength, and mental condition. During the day, hospitalized patients should perform the exercises alone by following a guided self-assessment for people with an acquired disability, which should be administered by physiotherapists or kinesiologists. This progressive approach as well as the known effects of general well-being can help keep patients busy and attenuate feelings of depression due to complete immobility. Regaining self-mobility can result in the patient acquiring better self-esteem and a strong response to depression.

Pneumonia, a severe complication of the virus, has been shown to induce cognitive decline due to sustained hypoxia [38,39]. Moreover, pneumonia patients were found to possess high levels of pro-inflammatory cytokines, leading to neuroinflammation and neurodegeneration [40]. Therefore, the positive influence of physical activity on cognitive performance is fundamental to accelerate the subsequent full recovery of COVID-19 patients. In fact, different studies have demonstrated that physical exercise enhances the neuronal activity and hippocampal neurogenesis essential for cognition [41,42]. Furthermore, adapted physical activity for COVID-19 patients represents a key psychological support. Exercise stimulates the cholinergic, dopaminergic, and serotonergic systems, enhancing mood by reducing depression, anxiety, and panic attacks [6,34]. In light of these positive effects on mental and psychological health, adapted or tailor-made exercises in COVID-19-affected individuals should be considered.

## 4. Adapted Physical Activity Program Post COVID-19 Infection

Once completely well, to maintain mental and physical well-being, it is vital for infected individuals to gradually resume physical activity and exercise with an adapted or tailor-made home-based exercise program administered by a sport scientist. The goal is to return to pre-infection levels of fitness. In this phase, the effects of physical activity on the brain trigger systemic influences on the entire body. Moreover, exercise promotes the release of endorphins, which enhance psychological well-being, favoring faster recovery and a return to normal life. Beyond conventional exercises to improve physical conditions, different activities are recommended to enhance psychological well-being, such as listening to music, reading or listening to a book, watching TV, playing cards, table games, and the utilization of “exergames” (i.e., active video games). These activities allow patients to keep busy, reducing depression. In particular, the use of exergames can positively affect motivation and self-efficacy by inducing physical activity practice [43]. Exergames use action and motion sensors, which allow the patient to be physically active, simulating several sport types, such as cycling, running, walking, rowing, and swimming. Moreover, the patient can also play exergames with a partner, favoring interaction and communication between them [44]. Another potentially beneficial activity during the patient recovery could be yoga. The practice of this discipline promotes endogenous melatonin secretion, positively affecting sleep quality, anxiety, and depressive disorders [45].

To allow the complete recovery of individuals, an interesting approach with several therapeutic benefits is Nordic walking. This activity is typically carried out in “healthy” environments, such as mountain, sea, and countryside settings, and is suitable for people of all ages. Nordic walking is useful for adapted motor re-education, especially for COVID-19 patients who have developed respiratory, metabolic, cardiovascular, and walking problems. Through the use of a specific pair of poles, Nordic walking engages the upper body muscles, and relative to normal walking would increase the overall energy expenditure [46]. Furthermore, since poles are held in both hands, the knees and joints are subjected to less stress; therefore, Nordic walking might be recommended in degenerative cartilage disorders, such as osteoarthritis [47], as it improves motor function and strength [48]. Interestingly, Nordic walking e-poles developed by Gabel, the Italian leading manufacturer in this area, are able to acquire the primary parameters that characterize the proper movement technique, providing feedback regarding the patient’s performance and assisted walking. Nordic walking strengthens cognitive function, attention, and executive functions by positively affecting patient quality-of-life [49]. It is noteworthy that Nordic walking also exerts a positive effect on an individual’s psychological well-being. Compared to normal walking, a previous study showed that this discipline elicited significant psychological improvements, ameliorating depression and sleep disturbance [50].

## 5. Conclusions

The SARS-CoV-2 virus represents the major societal challenge, with important repercussions for people’s mental and physical health. The beneficial effects of physical exercise in improving quality of life and well-being have been extensively documented. An adapted physical activity program may represent an important factor to prevent COVID-19 infection, as well as a useful complementary tool to improve the physical and psychological outcomes of COVID-19-affected patients. A suitable exercise program may strengthen the respiratory system, providing immune protection in the long term and reducing treatment costs. Furthermore, in the post-infection phase, an adapted or tailor-made home-based exercise program ensures a faster return to pre-infection fitness by enhancing self-esteem and resilience to stress and reducing anxiety and depression.
